# Emergence of the lymphogranuloma venereum L2c genovariant, Hungary, 2012 to 2016 

**DOI:** 10.2807/1560-7917.ES.2017.22.5.30455

**Published:** 2017-02-02

**Authors:** Fruzsina Petrovay, Eszter Balla, Tímea Erdősi

**Affiliations:** 1 Department of Bacteriology II., National Centre for Epidemiology, Budapest, Hungary; 2 Department of Phage and Molecular Typing, National Centre for Epidemiology, Budapest, Hungary

**Keywords:** Chlamydia trachomatis, lymphogranuloma venereum (LGV), men who have sex with men (MSM), L2c genovariant

## Abstract

In eastern Europe, few countries have so far reported laboratory-confirmed cases of lymphogranuloma venereum (LGV). Here we describe 22 LGV cases in men who have sex with men (MSM) detected in Hungary from November 2012 to July 2016. Sequence analyses show that 16 of these 22 cases were affected by the L2c genovariant, with from 2012 to 2014, one LGV L2c case detected per year, followed by seven cases in 2015 and six up to July 2016. Of the 16 total L2c LGV cases, 10 had severe haemorrhagic proctitis. These findings are concerning as cases with this new genovariant among MSM have not been frequently reported in Europe to date. More research is needed to assess the spread of the L2c genovariant and its potential association with virulence and severe clinical manifestation.

## Introduction

Among men having sex with men (MSM), lymphogranuloma venereum (LGV) causing proctitis and anorectal ulceration has been a re-emerging sexually transmitted disease in Europe since 2003, and many western and northern European countries have reported cases regularly [1,2]. In southern and eastern Europe however, only a few cases have been documented so far, the first in the Czech Republic in 2010, followed by Hungary in 2012 and Slovenia in 2015 [3-5]. In the Czech Republic, the number of detected cases has steadily increased since the initial identification [6].

The current LGV epidemic in western Europe is caused by the L2 biovar of *Chlamydia trachomatis* with the predominance of the L2b genovariant [7,8]. While most infections have occurred anorectally in human immunodeficiency virus (HIV)-positive MSM who have a high-risk behaviour, only few urethral infections have been reported [9]. Moreover a new LGV genovariant, designated L2c, which is a recombinant of L2 and D strains, has also been identified, but only a few persons affected by it have been documented so far [5,10]. 

The L2c genovariant was originally isolated from a case with severe haemorrhagic proctitis, showing a unique clinical pattern, and was described as a hypervirulent LGV strain developing cytotoxic phenotype in culture [10]. Despite this, there is no evidence in the literature confirming the association of this genovariant with more severe clinical manifestations. So far, the L2c genovariant is relatively rarely reported and only its emergence is mentioned in the updated European guideline on the management of LGV [11]. There are moreover no epidemiological data on its prevalence in western European countries. 

In eastern Europe testing for LGV has only recently been implemented so detection of cases has just begun. There are no published data about the LGV genotypes identified in the Czech Republic and only one confirmed L2c genotype was reported from Slovenia in 2015 [3,5,6]. In another neighbouring country, Austria, a high rate of non L2b variants among reported LGV cases (7/15) was observed in 2008, however sequenced-based identification was not performed at that time [12].

In this report we present findings of laboratory-confirmed LGV cases in Hungary from November 2012 through June 2016 as well as the sequence analysis results of isolates collected.

## Methods

### Clinical specimens

Since November 2012, i.e. after the first laboratory-confirmed LGV case in Hungary [4], 21 further cases have been investigated and confirmed as LGV in the Bacterial STI Reference Laboratory of the National Centre for Epidemiology, Budapest. Swab samples from the anus, urethra, a penile ulcer or an inguinal bubo as well as native blood samples were taken by venereologists of various genitourinary medicine (GUM) clinics and sexually transmitted infection (STI) outpatient wards in Budapest. While in the capital, these clinics and wards offer their services to patients from the whole country. The physicians provided clinical information based on the patients’ statements and on the respective physical examinations. Medical history (HIV serostatus) was also included.

### Laboratory investigations

LGV cases were laboratory-confirmed in a two-step protocol. Following DNA isolation, all samples were screened by an in-house *C. trachomatis* PCR targeting the plasmid gene. When the PCR was positive for *C. trachomatis* genetic material, LGV infection was confirmed with a *pmp*H real-time PCR which differentiates the L serovars from the other urogenital serovars of *C. trachomatis* [13]. The samples were then stored at − 20 °C until further identification of the genovariants. A fragment of the *omp*1 gene (ca 1,070 bp) was subsequently PCR amplified using a previously described protocol [14]. In order to genotype, a partial sequence from this 1,070 bp amplicon (ca 900bp) was obtained by sequencing, and aligned to reference sequences from GenBank representing different LGV variants: L2a (GenBank accession number: AF30485); L2(AM884176); L2b(AM884177); L2c (NC_015744); L2d (EF460797); L2e (EF460798); L2f (EU676181); and L2g (EU676180).

Additionally all anogenital samples were screened by PCR for *Neisseria gonorrhoeae and Treponema pallidum*. *T. pallidum* serology was performed simultaneously.

## Results

### Patients’ characteristics

LGV was confirmed in altogether 22 cases between 2012 and 2016. The distribution of the 22 LGV cases in time, after the first identified case in 2012, was as follows: two LGV in 2013, three in 2014, eight in 2015 and eight until July 2016 respectively.

The age distribution showed that the 25–34 years age group included most cases (10/22) followed by the 35–44 years age group with seven cases (Table).

**Table:** Characteristics of 22 laboratory-confirmed lymphogranuloma venereum cases, Hungary, 2012–2016

Characteristics	Sequenced genovariants^a^ Number of cases			Total
	L2c	L2b	L2 variant^b^	
	16	3	2	22^a^
**Age group (years)**				
15–24	3	0	0	3
25–34	8	1	1	10
35–44^a^	4	2	0	7
45–54	1	0	1	2
**Localisation of the LGV infection**				
Rectum	10	3	1	14
Urethra^a^	1	0	1	3
Penile ulcer	4	0	0	4
Inguinal lymph node	1	0	0	1
HIV status**^c^**				
Positive^a^	11	3	2	17
Negative	4	0	0	4
**Co-infection**				
Gonorrhoea	5	1	1	7
Syphilis	1	0	0	1
**Reinfection**				
Yes	2	0	0	2
No^a^	14	3	2	20

HIV: human immunodeficiency virus; LGV: lymphogranuloma venereum.

^a^ The genovariant could not be sequenced and identified in one case.

^b^ These variants could not be identified using the previously known reference sequences. Their sequences were identical to a sequence with GenBank accession number JX971936.1 [17] and, according to a recent article [15], the variants were designated L2bV1.

^c^ HIV-status was not available in one case.

Of the 22 cases, 13 came from Budapest or from the neighbouring cities of Pest county. The remainder were from various locations in the rest of Hungary. Nine cases mentioned having unprotected sexual contact with one or more foreign partners and/or visiting abroad recently. A total of 16 cases could not identify either their respective contact(s) or the possible site/time of infection. Only two cases were identified through contact tracing, one of them having serious proctitis, the other with an asymptomatic urethral carriage of the pathogen. The distribution of the 22 positive samples was as follows: 14 anal swabs, three urethral swabs, four penile swabs taken from the ulcerative lesions and one inguinal aspirate. Proctitis was the most common clinical manifestation observed among the cases (n=14). Other clinical symptoms included inguinal lymphadenopathy (n=6 cases), penile erosion (n=5), peri-anal ulceration (n=3), and urethral discharge (n=3). In two cases, patients suffering from rectal symptoms had been misdiagnosed and treated for irritable bowel disease (IBD) before the correct diagnosis of LGV was established.

### Laboratory findings

Sequence analysis of the LGV isolates showed that 16 of the sequenced samples (n = 21) were identical to L2c, three were identical to L2b, while two sequences could not be identified using the previously known reference sequences. These were designated L2bV1 according to a recent article [15]. The sequencing of one isolate failed in spite of repeated attempts because of low copy number of DNA. Distribution of cases with the L2c genovariant in time was as follows: one identified in 2012, in 2013 as well as in 2014; seven in 2015 and six until July 2016. Three patients with LGV infection caused by the L2c genovariant reported a recent visit to western European countries. Severe haemorrhagic proctitis was observed in 10 of the 16 LGV cases caused by the L2c genovariant. This particular manifestation was not seen in cases with the other genovariants.

Among the total 22 LGV cases, co-infection with *N. gonorrhoeae* was present in seven cases. *T. pallidum* DNA was detected in one case, however syphilis serology revealed that six patients suffered from a recent infection, nine had had previous syphilis infection, and only seven were seronegative. Concerning HIV status, 17 of 21 cases with this information available were HIV-positive (Table). Among severe proctitis cases (n=10), all of them affected by the L2c genovariant, co-infection rates were as follows: two patients’ serological results suggested recent syphilis, one patient was co-infected with gonorrhea while another two patients showed both characteristics, i.e. possible co-infection with *N. gonorrhoeae* and *T. pallidum* (the latter was assumed by high reactive serological results). 

## Discussion

As in the Czech Republic and countries of western Europe, we have noted an increasing number of LGV cases in Hungary after identification of the first case, which took place in November 2012. This increase may be due to the growing awareness of LGV among venereologists, which plays an important role in improved detection. Our STI laboratory is the only referral centre in Hungary that does confirmatory LGV testing and typing. Furthermore the institute’s task as the National Centre for Epidemiology, also includes organising courses and giving lectures about the situation of the STI surveillance and the current LGV status in Hungary. These efforts have likely led to the increase of detected LGV cases.

There are no specialised MSM clinics in Hungary where routine LGV screening is offered to any patients. Symptomatic MSM patients usually attend GUM clinics or consult private venereologists or proctologists. They are not necessarily screened for LGV, however multiple site screening for bacterial STIs (chlamydia, gonorrhea, syphilis) is frequently performed. When LGV is diagnosed, the LGV status of asymptomatic partners can also be revealed in case of successful contact tracing. It is notable that a number of LGV cases in our study had visited abroad (e.g. Germany, Spain, the Netherlands) and had had foreign sexual partners besides multiple anonymous contacts and unprotected sexual practices. Difficulties in partner notification made it almost impossible to identify the actual sources of infection, whether in Budapest, the rest of Hungary or abroad.

Patient characteristics in our report were comparable to those reported by western European countries where LGV has been screened routinely for almost a decade, as well as to those in the recent report from the Czech Republic [6,11]. In our study, LGV was not frequent (n=3) in the youngest MSM age groups (≤24 years) and most patients (10/22) were 25–34 years of age. The majority (n=17) of LGV patients were HIV-positive. Moreover many (9/22) had a concomitant STI and syphilis serology showed reactivity in 14 patients. *C. trachomatis* was detected mainly (n=14) from the rectum of patients with proctitis. Interestingly however, in seven cases it was isolated from the urethra or a penile ulcer with symptoms of urethral discharge or ulceration, and in one case from an inguinal bubo (Table).

The relatively high rate of urethral/penile LGV positivity (7/22) among our LGV patients contrasts with data reported in the literature and may be explained by a great number of misdiagnosed rectal infections where patients were not tested for LGV at all [16]. One of the LGV positive urethral samples originated from an asymptomatic contact of a previously diagnosed rectal LGV patient. For this contact, the rectal sample had proved LGV negative, which points to the crucial roles of contact tracing, and testing for extra-anal regions as well. Clinically, penile ulcers may be misdiagnosed as primary syphilis so multiple testing for bacteria causing STIs including LGV is also essential in high risk MSMs. This allows detecting possible co-infections as well.

Based on the current European Guideline, anorectal *C. trachomatis* screening should be performed routinely in MSM reporting risky sexual behaviour [11]. In case of patients’ symptomatic urethritis or ulcerative genital lesions the screening should be also extended to these extra-anal anatomic sites, as a rectal sample alone may be negative for LGV resulting in a misdiagnosis.

In contrast to western Europe, where the L2b genovariant has been reported to predominate, the sequence analysis in our study in Hungary revealed that the majority (n=16) of the LGV patients were caused by the recently described L2c genovariant. Conversely, the L2b genovariant was found in only three cases [7,11]. To our knowledge there is no report to date describing such a high rate of the L2c genovariant in Europe. In our patient population, all four HIV-negative patients had an LGV infection with the L2c genovariant and interestingly this genovariant was present in six of eight patients having extra-anal manifestations (Table).

These findings suggest that the new genovariant L2c of *C. trachomatis* LGV strains may have started to spread in Europe, hence continued analyses of sequences from further detected LGV cases are needed to confirm this. Genotyping is a useful tool for identifying outbreaks, confirming epidemiological links, revealing new variants and their role in certain groups of patients and in disease severity.

We found that half of the patients with severe L2c proctitis suffered from recent syphilis or from acute gonorrhea or both. These STI pathogens may contribute to the haemorrhagic symptoms and may cause more severe inflammation of the rectal mucosa.

It is noteworthy that two isolates in our study could not be identified as a formerly known L2 genovariant. Instead, their sequence analysis showed identity with an LGV variant sequence described in a Spanish study (GenBank accession number JX971936.1). This variant was presumably introduced to Europe through a South American-Spanish route and besides Spain it was also reported in France by Peuchant et al., where it was designated L2bV1 [15,17].

Our data have some limitations. Due to the high proportion of residents living in the capital area who represented more than half of the cases, our observations do not allow us to conclude as to the overall characteristics of all Hungarian patients affected by LGV. 

Moreover, a number of LGV cases are likely to remain undetected at this time, as LGV is still underdiagnosed in Hungary, due to most clinicians being neither aware of the condition itself, nor of the targeted diagnostic possibilities. As part of our routine screening for multiple bacteria responsible for STIs of venereological patients, we tested all patients with clinically suspicious conditions for *C. trachomatis*, and when LGV was found among positive samples, this laboratory result was often unexpected for the clinicians. As LGV diagnostic tests are only available at our laboratory in Budapest, this may also affect the willingness to send samples from other cities for testing. In addition, many private venereologists or proctologists send anogenital samples of symptomatic MSM patients to other laboratories where only the presence of *C. trachomatis* DNA is revealed without further confirmatory typing. Hence the patients might receive treatment without having the correct diagnosis. In 2015, four LGV cases that had occurred earlier that year were diagnosed retrospectively with LGV, when *C. trachomatis* DNA samples were sent to our institute from another laboratory after clinicians contacted us for further investigation. These LGV cases would have surely remained otherwise unnoticed. Moreover two patients had been misdiagnosed and treated for IBD before the correct diagnosis of LGV was established. Therefore informing and educating gastroenterologists, especially proctologists about this condition in the MSM population would also be essential.

Another limitation of this study is that we do not have any precise data regarding the actual number of chlamydial infections in the country and thus the LGV rate among detected chlamydial infections could not be derived. 

Finally, the lack of anamnestic data has to be mentioned, as only six patients provided information regarding their possible contacts or infections sites. This makes it extremely difficult to evaluate the epidemiological situation and to assess the source of L2c genovariants.

### Conclusion

LGV among MSM has already been spreading in Hungary since 2012 and based on Czech and Slovenian reports it is likely present in other southern or eastern European countries as well. Therefore intensified testing should be considered and increasing awareness among clinicians should be actively promoted. Venereologists and proctologists in particular should be informed of the presence of LGV in the MSM population, as well as on the typical symptoms, so that they can recognise the condition and be involved in a more effective, targeted surveillance system. 

Public health interventions are needed to inform the MSM population about LGV by online channels or by social/health services. Our laboratory took part in an anonymous STI screening campaign including LGV that was performed in 2015 for 500 MSM persons (results are being processed). These screening efforts may result in a growing number of identified cases, a better understanding of the prevalence of different genovars and less misdiagnosis and wrong treatment of the infection. The virtual dominance of the L2c genovariant among Hungarian LGV cases warrants this variant to be further investigated in Europe.
