# Addendum for Euro Surveill. 2017; 22(5)

**DOI:** 10.2807/1560-7917.ES.2017.22.26.30564

**Published:** 2017-06-29

**Authors:** Eurosurveillance editorial team

**Affiliations:** 1 European Centre for Disease Prevention and Control, Stockholm, Sweden

In the article Emergence of the lymphogranuloma venereum l2c genovariant, Hungary, 2012 to 2016 by F Petrovay et al., published on 02 February 2017, the addendum shown below was added on 29 June 2017, at the request of the authors.

The predominating *Chlamydia trachomatis* strains causing lymphogranuloma venereum (LGV) described in the article, were initially identified as L2c genovariants. They were further investigated by the LGV Genotype Dynamics Study Group at the University Hospital Basel. Analyses showed the strains possess an L2 ompA-genotype (identical to accession number AM884176), and L2b pmpH-genotype [1]. Thus they share features with new variants of LGV described recently [2]. The majority of Hungarian LGV isolates appear to be this new genovariant. Based on ompA sequence analysis alone, one cannot reliably distinguish the classic and the novel L2 strains. Currently there is no distinguishing nomenclature for these strains.
